# Management of delayed corneal epithelial healing after refractive surgery: five case reports

**DOI:** 10.3389/fmed.2025.1517403

**Published:** 2025-03-04

**Authors:** Chunxiao Yan, Lin Jin, Qiaosi Zhang, Xiaoyu Liu, Taorui Yu, Fangkun Zhao, Yanan Mu, Jun Xu, Lijun Zhang

**Affiliations:** ^1^Department of Ophthalmology, The Third People’s Hospital of Dalian, Dalian Municipal Eye Hospital, Dalian Municipal Cancer Hospital, Liaoning Provincial Key Laboratory of Cornea and Ocular Surface Diseases, Liaoning Provincial Optometry Technology Engineering Research Center, National Clinical Research Center for Eye Diseases, Dalian, Liaoning, China; ^2^Dalian Medical University, Dalian, Liaoning, China; ^3^Department of Ophthalmology, The Fourth Affiliated Hospital of China Medical University, Shenyang, Liaoning, China

**Keywords:** delayed corneal epithelial healing, refractive surgery, corneal virus infection, persistent epithelial defects, trans-PRK

## Abstract

**Background:**

Transepithelial photorefractive keratectomy using Smart Pulse Technology (SPT-TransPRK) is currently the leading method for superficial refractive surgery, offering advantages such as a non-contact procedure, shorter operation times, and excellent patient cooperation. Laser ablation of the corneal epithelium, Bowman’s membrane, and the stroma can effectively correct refractive errors. Thus, the complete healing of the corneal epithelium post-surgery is essential for ensuring good vision. Refractive surgeons should enhance their understanding of corneal wound healing mechanisms and focus on the repair of the corneal epithelium following refractive surgery to ensure the quality of visual health of patients.

**Case presentation:**

A total of five patients experienced varying degrees of delayed corneal epithelial healing following refractive surgery. In Case 1, unhealthy corneal epithelial debris was removed, and ophthalmic ointment was applied to cover the eyes instead of using bandage contact lenses (BCLs) to reconstruct the corneal epithelial barrier. This approach was also successfully implemented in Case 2. Furthermore, amniotic membrane transplantation (AMT) can quickly establish a corneal barrier and promote corneal epithelial regeneration, especially in cases of extensive corneal epithelial detachment. The remaining three patients were suspected of having corneal viral infections based on their medical history and the observation of corneal pathology using a slit lamp microscope. To prevent further infection and promote regeneration, topical steroid drops were discontinued early, and topical antiviral and corneal epithelial regeneration medications were administered alongside systemic antiviral therapy. Steroid drops were resumed after corneal epithelial healing to effectively prevent post-refractive haze.

**Conclusion:**

Delays in corneal epithelial healing after refractive surgery should be taken seriously. BCLs, steroids, and both topical and systemic antiviral therapies should be properly utilized when there is a delay in corneal epithelial healing.

## Introduction

Transepithelial photorefractive keratectomy (TransPRK) was introduced in the late 1990s as an alternative to conventional photorefractive keratectomy (PRK) for corneal refractive surgery (1). This procedure was developed to replace traditional PRK and has evolved with advancements in technology and equipment. One notable advancement is the integration of transepithelial photorefractive keratectomy with Smart Pulse Technology (SPT-TransPRK), which has become the standard approach for personalized surface ablation. SPT allows for a more precise distribution of laser emissions in a fullerene 3D model, resulting in cleaner laser cut and a smoother post-ablative stromal bed surface. This enhanced precision supports the migration of corneal epithelial cells, potentially contributing to the rapid recovery of the corneal epithelium and the reduction of postoperative ocular surface pain following SPT-TransPRK (2, 3). SPT-TransPRK is widely recognized as a safe and effective surgical procedure (4, 5). Currently, the average number of refractive surgery cases in China is approximately 1 million per year (6). Despite the widely recognized safety, efficacy, and predictability of photorefractive keratectomy, clinicians must remain vigilant regarding the overall risks associated with refractive surgery. Delayed corneal epithelial healing after SPT-TransPRK is rare; however, the factors influencing postoperative corneal epithelial healing are complex. This study reports five cases of delayed corneal epithelial healing after SPT-TransPRK that occurred between 2019 and 2023. We aim to summarize the treatment plan for this condition by analyzing the etiology of persistent epithelial defects (PED) in different individuals. This study aims to raise awareness among refractive surgeons regarding the importance of thorough preoperative screening and treatment to enhance the overall safety and effectiveness of refractive surgery.

## Case presentation

### Case 1

**Figure 1:**
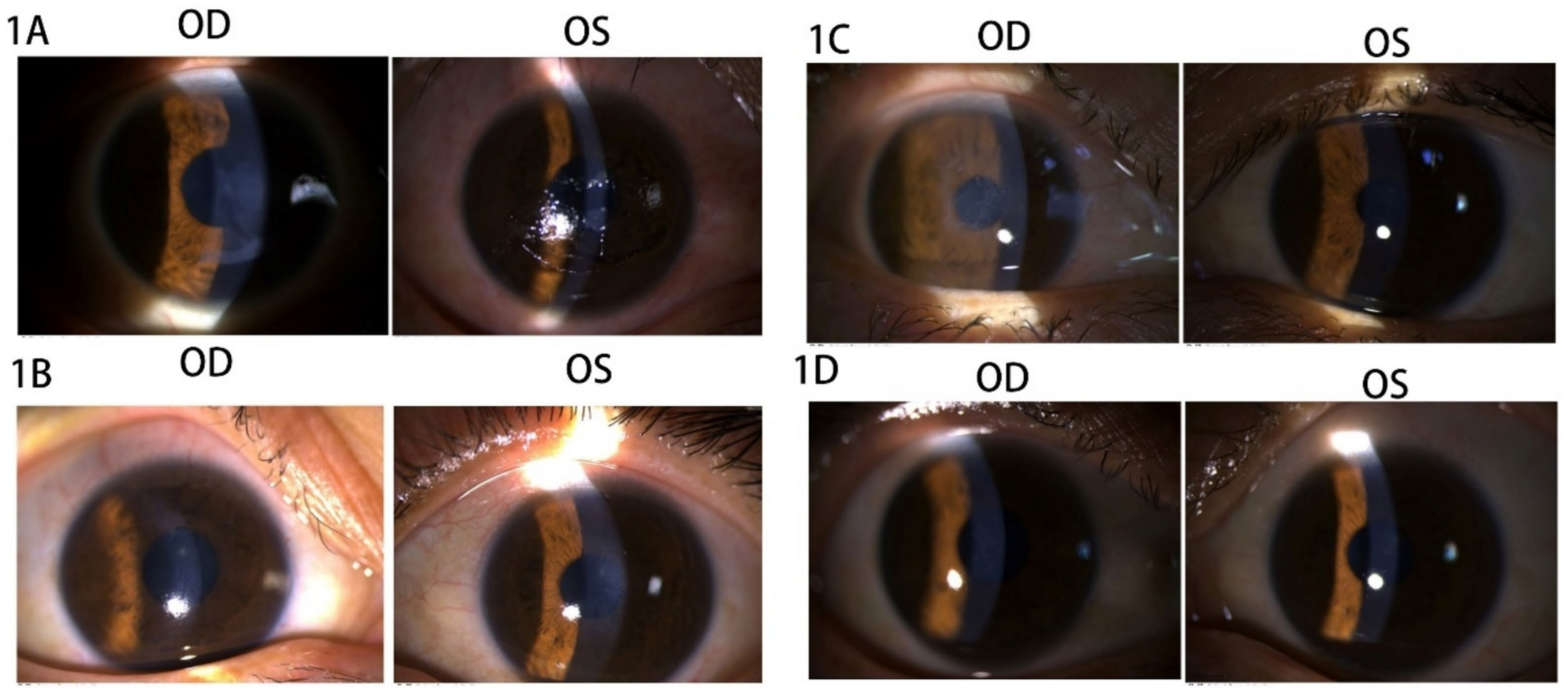
Images of the anterior segment of the cornea in Case 1. **(A)** The corneal epithelium was not completely healed in both eyes during 1 week. **(B)** The corneal epithelial regeneration was not been improved within 1 week. **(C)** The central corneal epithelium defect of 3 mm in diameter was observed in the right eye at 3 weeks after the surgery. **(D)** At 4 weeks postoperatively, the bilateral cornea returned to be transparent.

The patient was a 32-year-old woman who underwent cycloplegic refraction, resulting in −2.25 –0.75 × 164 in the right eye (OD) and − 2.25 –1.25 × 15 in the left eye (OS). In 2018, SPT-TransPRK was performed after a thorough assessment that ruled out contraindications (The details of contraindications are documented in the Supplementary material section). The patient reported no prior history of herpes simplex virus infection. Preoperatively, anti-inflammatory eye drops were administered, and bandage contact lenses (BCLs) were fitted in both eyes. Postoperatively, anti-inflammatory and anti-infective eye drops were used (The details of medical guidance are provided in the Supplementary material section). During the 1-week postoperative assessment, the patient’s uncorrected vision acuity (UCVA) was 20/30 in the right eye and 20/25 in the left eye. A delay in the complete healing of the corneal epithelium was observed in both eyes (see Figure 1A), leading to a diagnosis of bilateral delayed corneal epithelial healing. Accumulation of corneal epithelial debris was noted beneath the BCL in the right eye, necessitating its replacement with a new lens; the original BCL for the left eye remained in place. However, there was no improvement in bilateral corneal epithelial regeneration after 1 week (see Figure 1B). Following the replacement of the BCL in the right eye, the left BCL was removed, and treatment commenced with 0.3% sodium hyaluronate eye drops administered once per hour, 0.5% levofloxacin eye drops three times a day, loteprednol etabonate ophthalmic suspension three times a day, and carbomer eye gel once nightly. Three weeks post-surgery, the right eye exhibited photophobia and had a UCVA of 20/25. A central corneal epithelial defect measuring 3 mm in diameter was observed in the right eye (see Figure 1C). Given that the BCL had been worn consistently in the right eye, it was hypothesized that its presence may have interfered with epithelial regeneration. Consequently, the BCL was removed, and the eye was treated with tobramycin dexamethasone ophthalmic ointment, gatifloxacin eye gel, and deproteinized calf blood extract eye gel, applied three times daily. At the four-week postoperative evaluation, both corneas were transparent, and the UCVA improved to 20/20 for both eyes (OU) (refer to Figure 1D).

### Case 2

**Figure 2:**
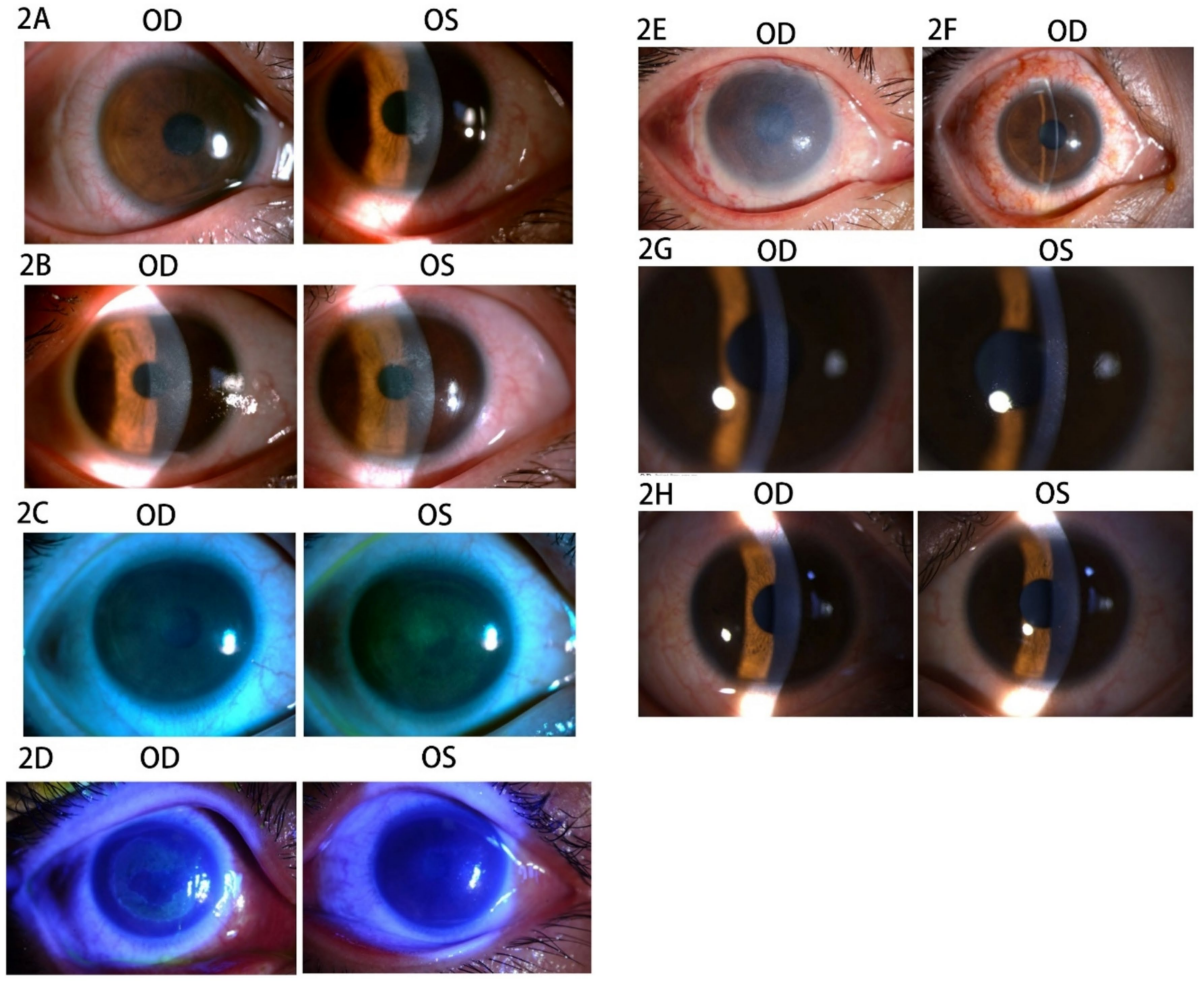
Images of the anterior segment of the cornea in Case 2. **(A)** The corneal epithelium showed slight punctate defects in the right eye, and the epithelium debris accumulated in the central cornea in the left eye. **(B)** The punctate erosion of corneal epithelium was observed in both eyes. **(C)** The sodium fluorescein staining revealed diffuse staining in both eyes. **(D)** The corneal epithelium of the left eye was visibly repaired, whereas the right eye exhibited extensive epithelial erosion after 2 days. **(E)** The amniotic membrane in the right eye. **(F)** The stitches were removed at 2 weeks after the AMT. **(G)** The cornea was transparent at 5 months after SPT-TransPRK. **(H)** The cornea was transparent at 9 months after SPT-TransPRK.

The patient was a 30-year-old woman with a cycloplegic refraction of −4.25 -0.75 × 88 in the right eye (OD) and − 4.25 -0.25 × 76 in the left eye (OS). She had a 12-year history of using soft corneal contact lenses (SCLs), which were removed 1 month prior to surgery. In 2018, SPT-TransPRK was performed after ruling out any contraindications. Details regarding contraindications, her history of herpes simplex virus infection, and the preoperative and postoperative management are provided in Case 1. The BCLs were removed 1 week postoperatively due to epithelial healing. However, recurrent corneal epithelial defects frequently occurred in both eyes within 2 months following surgery. The patient reported worsened foreign body sensations, pain, and photophobia in both eyes. An exam revealed slight punctate defects in the corneal epithelium of the right eye, which had a UCVA of 20/20. In the left eye, epithelial debris accumulated in the central cornea, resulting in a UCVA of 20/40 (Figure 2A). Consequently, the patient underwent corneal debridement in the left eye, and new BCLs were prescribed for both eyes. Postoperative care included routine medication. After 2 weeks, punctate erosions of the corneal epithelium were noted in both eyes, with UCVA of 20/32 in the right eye and 20/25 in the left eye (Figure 2B). The patient was advised to apply deproteinized calf blood extract eye gel along with tobramycin-dexamethasone ophthalmic ointment three times daily after removing the BCLs.

Nevertheless, there was no significant improvement after 1 week. The patient was instructed to use an autologous serum for 1 week, during which the UCVA decreased to 20/40 OU, and sodium fluorescein staining revealed diffuse staining in both eyes (Figure 2C). Deproteinized calf blood extract eye gel and tobramycin-dexamethasone ophthalmic ointment were applied three times daily to both eyes during hospitalization. After 2 days, the corneal epithelium of the left eye showed visible repair, whereas the right eye displayed extensive epithelial erosion (Figure 2D). The patient then underwent amniotic membrane transplantation (AMT) in the right eye to restore the corneal epithelial barrier (Figure 2E). After AMT, tobramycin dexamethasone ophthalmic ointment was administered once at night, along with ofloxacin ophthalmic ointment three times daily and recombinant bovine basic fibroblast growth factor eye gel (rb-bFGF) four times daily in the right eye. The stitches were removed 2 weeks post-AMT, and the corneal epithelial healing was completed, achieving a UCVA of 20/25 OU (Figure 2F). At 5 and 9 months after SPT-TransPRK, the patient’s UCVA was 20/20 OU, and the cornea appeared transparent (Figures 2G,H).

### Case 3

**Figure 3:**
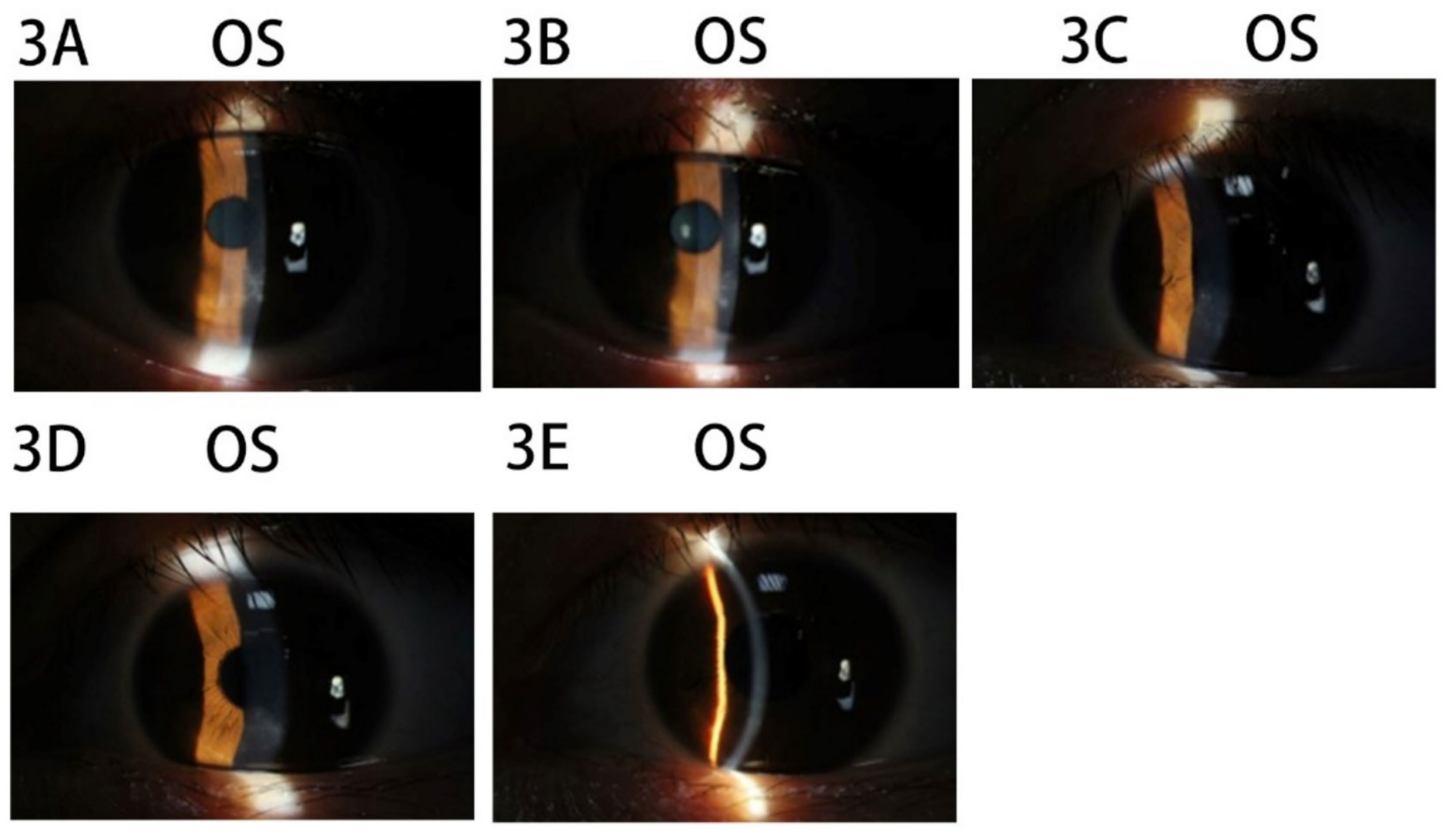
Images of the anterior segment of the cornea in Case 3. **(A)** The slightly opacity zone underneath epithelium below the pupil of the left eye. **(B)** The infiltration spot in the opacity zone in the left eye. **(C)** The infiltrated spots in the superficial stromal layer of the left cornea subsided. **(D)** The left corneal epithelium had healed and the subepithelial corneal opacity was alleviated. **(E)** The corneal epithelium was healed and stable.

The patient was a 21-year-old woman with a cycloplegic refraction of −6.00 –0.50 × 55 OD and −3.75 –0.50 × 174 OS. SPT-TransPRK was performed in 2020, and contraindications were excluded. Details regarding contraindications, history of herpes simplex virus infection, and the preoperative and postoperative management of the patient can be found in Case 1. On the 6th postoperative day, the corneal epithelium of the left eye had not healed well, and the BCL continued to be retained. By the 12th postoperative day, the epithelial surface of the left eye lacked smoothness. Consequently, BCLs were removed, and the patient was instructed to apply ganciclovir ophthalmic gel four times daily. One month postoperatively, a slight opacity zone was observed underneath the epithelium, below the pupil of the left eye (Figure 3A). At that point, the patient was advised to stop using steroid drops. After 1 week, the patient experienced photophobia in the left eye, and infiltration was noted in the opacity zone (Figure 3B). Follow-up history indicated that the patient frequently experienced a burning sensation in the left eye and applied cold air directly to it. Therefore, it was suspected that prolonged exposure to the air-conditioned environment could lead to a viral infection. The patient was instructed to apply eye gel three times daily (ganciclovir ophthalmic gel, gatifloxacin eye gel, and ofloxacin eye ointment) along with oral acyclovir once daily. After 1 week, the infiltrated spots in the superficial stromal layer of the left cornea subsided, and the previously mentioned antiviral therapy was continued for another week (Figure 3C). After an additional week, the left corneal epithelium had healed, and the subepithelial corneal opacity was alleviated (Figure 3D). Steroid medication was resumed, and antiviral treatment was discontinued. After three weeks, the corneal epithelium was healed and stable (Figure 3E).

### Case 4

**Figure 4:**
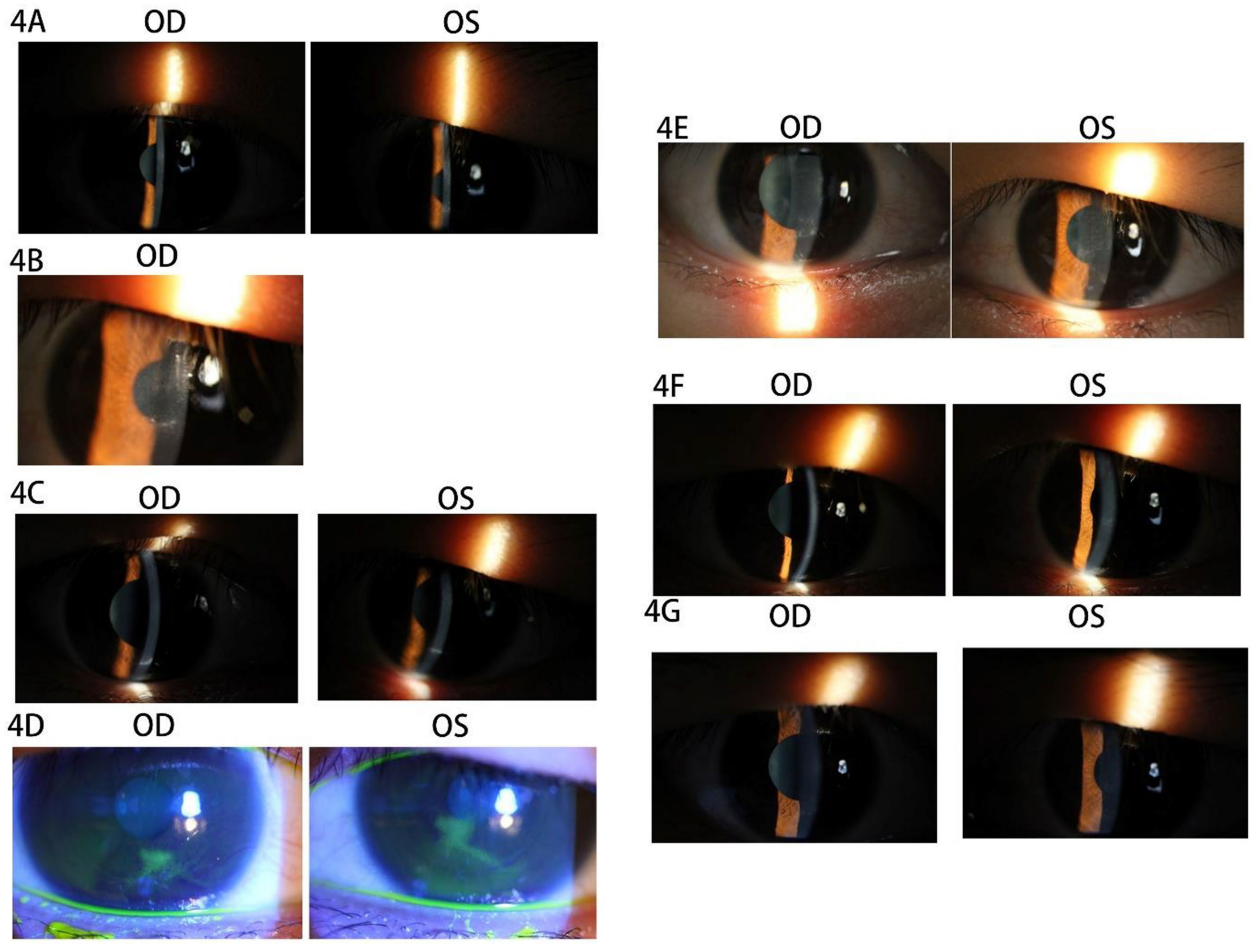
Images of the anterior segment of the cornea in Case 4. **(A)** Tthe corneal epithelial surface lacked smoothness in both eyes. **(B)** The accumulation of epithelial cell debris was below the pupil area. **(C)** The patient’s corneal epithelial cell debris accumulated the lower eyelid margin at 2 months postoperatively. **(D)** The dendritic staining of sodium fluorescein was orbserved after 1 week. **(E)** The rough corneal epithelia and haze grade 2 were observed in both eyes. **(F)** After 1 week, the corneal epithelium of both eyes healed well with haze grade 2. **(G)** The corneas of both eyes were transparent and smoothy at 4 months postoperatively.

The patient was a 27-year-old man with a cycloplegic refraction of −4.25 –0.25 × 75 OD and −3.00 –0.25 × 88 OS who had a history of wearing soft contact lenses (SCLs) for 9 years preoperatively, with a discontinuation period of 1 month. Bilateral eyelid strength was tight, with no contraindications. SPT-TransPRK was performed in 2019. The details of contraindications, the history of herpes simplex virus infection, and the preoperative and postoperative management of the patient were described in Case 1. At 10 days postoperatively, the patient returned to the clinic with a foreign body sensation and decreased vision (UCVA of 20/30 OU) in both eyes, and the corneal epithelial surface lacked smoothness (see Figure 4A). The patient was instructed to use vitamin A palmitate eye gel along with other conventional postoperative medications. Symptoms of eye irritation were alleviated, and the right corneal epithelium healed with a UCVA of 20/20.

The left corneal epithelial surface exhibited roughness, presenting with a UCVA of 20/30. Additionally, there was a noted accumulation of epithelial cell debris beneath the pupil area (refer to Figure 4B). The patient received treatment involving corneal debridement and a bandage contact lens (BCL) in the left eye, leading to complete healing within 1 week. However, 2 months postoperatively, the patient experienced a sudden decrease in UCVA (20/30 OU) alongside further accumulation of corneal epithelial cell debris at the lower eyelid margin (see Figure 4C). Notably, corneal epithelial debridement with BCL application was performed in both eyes during this period. One week subsequent to the procedure, the patient reported ocular irritation and demonstrated dendritic staining upon sodium fluorescein application (refer to Figure 4D). A suspected diagnosis of bilateral viral keratitis was made, potentially attributed to a preceding cold illness occurring 2 weeks prior. The patient was advised to discontinue the use of steroidal drops and to apply an eye gel regimen four times daily, which included ganciclovir ophthalmic gel, gatifloxacin eye gel, and deproteinized calf blood extract eye gel, in conjunction with oral acyclovir once per day. Following this treatment plan, the patient’s symptoms showed significant improvement, achieving a UCVA of 20/20 OU, alongside rough corneal epithelia and a haze grade of 2 in both eyes (see Figure 4E) after 1 week. Ocular topical and systemic antiviral therapy were continued for an additional 3 weeks. At the conclusion of this period, it was observed that the corneal epithelium of both eyes had healed adequately, maintaining a haze grade of 2 (refer to Figure 4F). The haze was subsequently treated with fluticasone 0.1% eye drops administered four times daily, following the cessation of antiviral therapy in both eyes. Notably, by 4 months postoperatively, the corneas of both eyes appeared transparent and smooth (refer to Figure 4G).

### Case 5

The patient was a 31-year-old man who presented with cycloplegic refraction measurements of −3.00 –0.25 × 3 OD and −1.50 –0.25 × 180 OS. He had a documented history of wearing soft contact lenses for 6 years, which were discontinued 3 months prior to the surgical intervention. Upon examination, the patient exhibited bilateral tight strength of the upper eyelids, and there were no noted contraindications. The specifics of any contraindications, along with the medical history of herpes simplex virus infection, as well as the preoperative and postoperative management strategies, are detailed in Case 1. The SPT-TransPRK procedure was conducted in 2022. The bilateral corneal diameters measured 11.1 mm OD and 11.3 mm OS, while the treatment optical zone and ablation zone dimensions were recorded at 6.8/7.97 mm OD and 7.1/8.04 mm OS, respectively. At 1 week postoperatively, the right corneal epithelium had healed appropriately, with UCVA recorded at 20/20. Conversely, the left corneal epithelial cell debris had accumulated beneath the bandage contact lens (BCL), with UCVA measuring 20/25.

**Figure 5:**
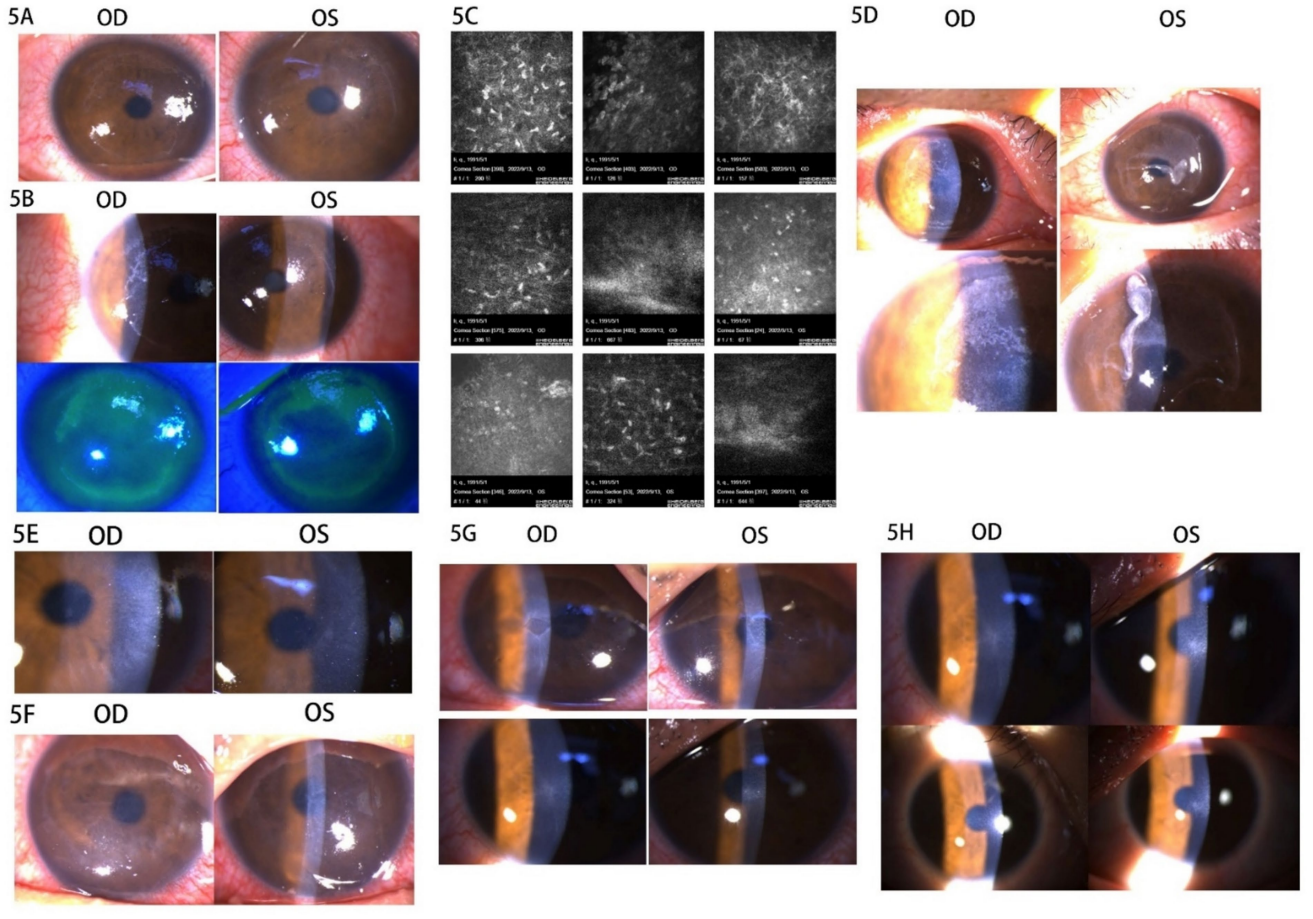
Images of the anterior segment of the cornea in Case 5. **(A)** The punctate infiltration of cornea was in both eyes. **(B)** The conjunctival ciliary congestion, suspected arborization infiltrative zone on the temporal side, and sodium fluorescein staining showed diffuse detachment in the corneal epithelium of both eyes. **(C)** The confocal microscopy images. **(D)** The bilateral corneal epithelium detachment with secretion and conjunctival ciliary congestion. **(E)** The right corneal epithelium was repaired with corneal edema and the left corneal epithelium was defective. **(F)** The cornea of both eyes healed no better than before. **(G)** The epithelial defect gradually decreased in 1 week. **(H)** The corneal epithelium had healed with haze grade of 0.5.

Based on the patient’s prior experience with the treatment, left cornea debridement was performed, and the BCL was replaced. The left corneal epithelium healed within a week. One month postoperatively, the corneal epithelium appeared normal, with objective refraction measurements of −2.00 –0.25 × 98 OD and −1.75 –0.75 × 44 OS, and UCVA measuring 20/25 OD and 20/30 OS. The steroid was switched to prednisolone acetate eye drops four times daily due to insufficient refractive correction following surgery, while the other medications remained unchanged. Four days later, the patient reported pain and secretion in both eyes, along with punctate infiltration of the cornea in both eyes, and was prescribed ganciclovir ophthalmic gel three times daily (Figure 5A). Two days later, the patient experienced acute sharp pain and secretion in both eyes, with conjunctival ciliary congestion and a suspected arborization infiltrative zone on the temporal side. Sodium fluorescein staining revealed diffuse detachment in the corneal epithelium of both eyes (Figure 5B). Confocal microscopy showed corneal epithelial cell edema, activated Langerhans cells in the basal layer, activated stromal cells, and inflammatory cells in the superficial stroma without evidence of fungal mycelium or amoebic cysts (Figure 5C). The patient had experienced a cold 4 days earlier and was also dealing with work stress, emotional anxiety, and poor nighttime rest. A diagnosis of corneal viral infection was suspected based on the patient’s history and ocular symptoms. The patient was advised to discontinue steroid use and to apply ganciclovir eye gel and gatifloxacin eye gel three times daily, along with systemic antiviral therapy (acyclovir 0.25 g + 0.9% physiological saline). One day later, the patient noted bilateral corneal epithelium detachment, accompanied by significant secretion and conjunctival ciliary congestion (Figure 5D). Bacterial and fungal culture tests were conducted on the secretions from both eyes, and levofloxacin drops were used to rinse both eyes. The BCL was applied, and ofloxacin ophthalmic gel was added to the previous regimen to prevent bacterial infection. After 1 day, the bilateral cornea appeared clear without infiltration, and bacterial culture results returned negative. The ofloxacin ophthalmic gel was discontinued and replaced with oral valacyclovir, which was taken twice daily in addition to the original treatment. The area of corneal epithelial defect decreased, with a small amount of secretion observed in the corneas of both eyes after 1 day.

The rb-bFGF eye gel was added to the previous treatment, and levofloxacin drops were administered eight times a day. One day later, the right corneal epithelium was repaired with corneal edema, while the left remained defective (Figure 5E). Levofloxacin drops were reduced to six times a day. After 2 days, the corneas of both eyes had not healed any better than before, so the deproteinized calf blood extract eye gel was added to the treatment (Figure 5F). The epithelial defect gradually decreased within 1 week (Figure 5G). The patient was instructed to continue the consolidated antiviral therapy for 2 weeks and to resume steroids. After 2 weeks, the corneal epithelium had healed with a haze grade of 0.5, and post-refractive medication was continued in both eyes (Figure 5H).

## Discussion

**Figure 6:**
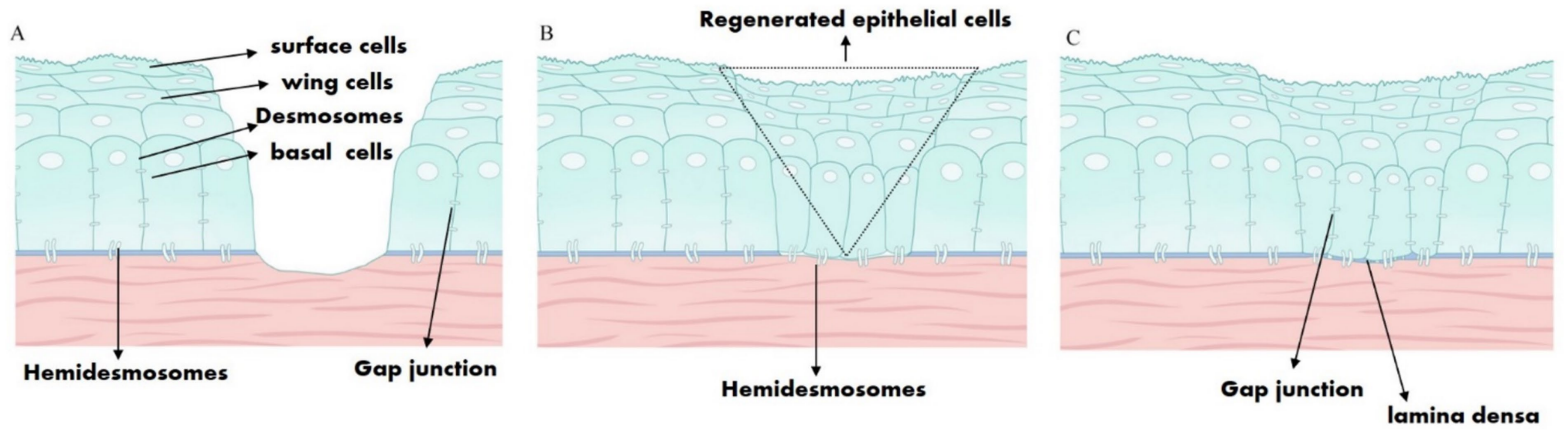
Schematic diagram illustrating the healing of corneal epithelial injury. **(A)** The corneal epithelial cells, hemidesmosomes, basement membrane, anchoring fibers, and anterior stroma were all damaged as a result of corneal epithelial injury. **(B)** Regenerated epithelial cells covered the defect, and hemidesmosomes were observed after 24–72 h. **(C)** The incomplete lamina densa of the basal lamina was observed.

The delayed healing of corneal epithelial tissue after refractive surgery is generally defined as corneal injury healing that lasts longer than 3 days. We reviewed relevant literature on corneal epithelial healing. Actin microfilaments within the corneal epithelium anchor to the corneal stroma through E-cadherin-mediated adhesion molecules, coordinating the migration of the epithelium (7). The adhesion complex of the corneal epithelium comprises hemidesmosomes, anchoring filaments, the lamina densa of the basal lamina, and anchoring fibers (8). Stock et al. (9) observed that hemidesmosomes became visible at the site of the corneal epithelial defect 24 h after injury, and an incomplete lamina densa of the basal lamina was noted beneath the hemidesmosome at 7 days post-injury in a guinea pig corneal epithelial injury model (Figure 6). When the proliferative epithelium covers the defect, basal cells begin to secrete laminin to initiate basement membrane reconstruction. These cells then differentiate into pterygoid epithelial cells and start migrating, creating a tight epithelial barrier with pterygoid epithelial cells linked by hemi-bridges (10). The basal cells receive biological signals from both the epithelium and keratocytes to stimulate the synthesis of basement membrane component proteins, which contribute to the stability of the basement membrane (11). Consequently, the lamina densa of the basal lamina is not fully repaired in the early postoperative period, leading to instability in the corneal epithelium and its inability to form a barrier. Persistent corneal epithelial defects may also involve fibrosis and keratocyte apoptosis (12, 13). Therefore, the healing of the corneal epithelium following refractive surgery should be a primary concern for refractive surgeons.

Refractive surgery is recognized as a relevant model of corneal injury, where inflammatory mediators trigger a cascade of biological responses (14). Initially, epithelial removal was viewed as a key factor influencing the healing of the corneal epithelium. Shapira et al. (15) indicated that alcohol-assisted or laser-assisted epithelial removal has a negligible effect on epithelial healing compared to the traditional mechanical method, which does not significantly impact long-term visual quality. In this study, the surgical procedure implemented in all cases was the SPT, characterized by a smoother stromal layer that facilitates corneal epithelial migration, as previously mentioned. Prompt corneal barrier reconstruction is crucial to prevent risk factors from infiltrating the tissue and avert subsequent pathological developments. Consequently, BCLs are widely used in superficial refractive surgery to alleviate postoperative ocular surface discomfort, enhance early postoperative visual acuity, expedite re-epithelialization, prolong drug contact with the ocular surface, and reduce the risk of postoperative infection (16). Notably, refractive surgeons must closely monitor the duration of BCL usage. Prolonged use of BCLs may lead to the accumulation of epithelial debris, tear proteins, and inflammatory mediators (17). Qiao’s research revealed that lysozyme deposition was detected on the inner surface of BCLs within 1 h (18). The patient referred to in Case 1 experienced the accumulation of corneal epithelial fragments beneath the BCL during the healing process, which necessitated a replacement BCL. Additionally, considering the extended use of the BCL without complete regeneration of the corneal epithelium, three types of eye ointment were utilized to protect the eyes as an alternative to BCLs.

Typically, refractive surgery patients receive SCLs to correct myopia. However, prolonged use of SCLs is often associated with various ocular surface complications, including infectious and non-infectious keratitis, allergic reactions, hypoxia, toxic reactions, mechanical damage, and dry eye (19, 20). Case 2 involved a patient with a 12-year history of BCL use prior to the procedure, which may lead to punctate detachment of the corneal epithelium, frequently occurring 2 months postoperatively. Therefore, upon reviewing this case, our team will focus on the preoperative corneal epithelial health of this specific group of patients in our future work. The amniotic membrane is considered a scaffold for epithelial cell migration after corneal injury and releases proteases, growth factors, and anti-inflammatory cytokines, which help prevent epithelial cell apoptosis (21). Moreover, no immune rejection occurred after transplantation (22). The corneal epithelium in Case 2 had not regenerated despite multiple medications, and since prolonged corneal epithelial defects could increase the risk of corneal infections, AMT was performed to quickly restore the corneal barrier, reducing the stimulation of the corneal stroma by inflammatory factors in the tear fluid and the formation of corneal haze.

Patients in cases 3–5 experienced varying degrees of viral infection due to delayed corneal epithelial healing. The causes of viral infection after refractive surgery included surgical injury, long-term postoperative steroid use, excimer laser activation of the virus, and reduced systemic immunity (23). Wu et al. (24) reported two cases of herpesvirus keratitis following SPT-TransPRK in 2022, which were treated with early topical antiviral drugs combined with systemic antiviral therapy. The surgeon diagnosed a “presumed viral infection” based on the history and corneal pathology without conducting PCR testing in this case series. Antiviral therapy had a positive effect on these three cases. The patient in Case 3 experienced a relatively mild corneal infection, and antiviral medications were administered when punctate infiltration was observed in the cornea. Steroids were suspended, effectively controlling the viral infection. In Case 4, the patient had a history of wearing SCLs for up to 9 years before the refractive surgery and presented with tight skin tension on both eyelids. As previously indicated, long-term use of SCLs could lead to abnormal epithelial metabolism. Similarly, the mechanical forces from the eyelids could directly affect the adhesion of the corneal epithelium (25). Therefore, these two factors may be potential reasons for delayed corneal epithelial healing. The selection of refractive surgery methods for patients with tight eyelid skin should be considered carefully to avoid delayed corneal epithelial healing. Furthermore, prolonged use of BCLs after surgery may not benefit corneal wound healing due to the combined effects of eyelid force and BCLs. The antiviral treatment strategy was noted in Case 3.

In Case 5, the surgery was designed to create a large treatment optical zone and ablation zone relative to the patient’s corneal diameter. The corneal nerve originates from the trigeminal ganglion, and the density of corneal nerves is more abundant beneath the basal cells of the corneal epithelium, which are arranged in a wheel-like pattern (26). Studies show that corneal nerves are often damaged after refractive surgery and regenerate incompletely within 1 year postoperatively (27). The corneal nerves directly influence the sensitivity and regeneration of the cornea (28). Therefore, rb-bFGF eye drops and deproteinized calf blood extract eye gel were incorporated into the treatment protocol alongside corneal debridement and anti-infection measures in Case 5. Treatment for this case primarily involved debridement, the application of antiviral and antibacterial drugs, and systemic antiviral therapy, all of which proved effective. Notably, when the cornea is suspected of being infected with a virus, steroids should be suspended promptly, and adequate doses of antiviral therapy should be initiated. The characteristics of all cases in the study are summarized in Table 1.

**Table 1:** The summary of all the cases in this study.

	Case 1	Case 2	Case 3	Case 4	Case 5
Age	32	30	21	27	31
Risk factor	BCL size	SCL wearing history	Presumed viral infection	Presumed viral infection	Presumed viral infection
Soft contact lens wear duration	None	12 years	None	9 years	6 years
Dry eye	None	None	None	None	None
Pre-op herpes simplex infection	None	None	None	None	None
Duration of PED	3 weeks	3 months	1 month	1 month	2 weeks
Etiology of PED	BCL size	Preoperative SCL wearing history	Presumed viral infection	Preoperative SCL wearing history, eyelids’ mechanical forces, and presumed viral infection	Presumed viral infection and the large ablation zone
The responded treatment	Replaced the BCLs with eye ointment wrapping	AMT	Topical and systemic application of ganciclovir earlier	Topical and systemic application of ganciclovir earlier	Suspension of steroid eye drops and topical and systemic application of ganciclovir earlier

Mitomycin C (MMC) at a concentration of 0.02% is widely used in refractive surgery as an anti-fibrotic agent that inhibits keratocyte fibrosis (29). However, Kremer’s case report indicated that the intraoperative use of MMC could result in delayed corneal epithelial healing (30), possibly due to MMC contacting the corneal limbus. In these five case reports, MMC was applied for only 20 s within the laser ablation zone, thereby excluding its role in the delayed healing observed.

In summary, delayed corneal epithelial healing after refractive surgery is rare. The study’s limitations primarily involve insufficient sample sizes; however, without prompt treatment, the quality of vision in post-refractive surgery patients can be significantly compromised. Therefore, we compiled all instances of delayed healing since the onset of refractive surgery at our medical center and shared our successful management experiences, focusing on etiological analysis and treatment strategies. In other words, when corneal epithelial healing is inadequate, a thorough review and analysis of the patient’s consultation information should be conducted to identify potential causes of delayed healing and determine additional treatment options.

## Data Availability

The original contributions featured in the study are included in the article and Supplementary material. Any further inquiries can be directed to the corresponding author.
