# Supplementary material for: Management of delayed corneal epithelial healing after refractive surgery: five case reports
Source: Front Med (Lausanne). 2025 Mar 4;12:1517403. doi: 10.3389/fmed.2025.1517403 (PMC11913852; doi:10.3389/fmed.2025.1517403)
Supplement: Supplementary file 1 [file Data_Sheet_1.docx]

**Trans-PRK surgical contraindication：**

1.The severe systemic autoimmune diseases, e.g. rheumatoid arthritis, diabetes.

2.The severe diseases of the ocular appendages, e.g. eyelid deformity, chronic dacryocystitis.

3.The severe ocular surface diseases, e.g. meibomian gland dysfunction, dry eye, keratoconus, active viral keratitis and corneal endothelial dystrophy.

4. Glaucoma, iritis, ciliary body inflammation and optic nerve disease.

5. High myopia combined with thin cornea and large pupil diameter.

6. People with unhealthy heart

**Guidance derived from“*Expert consensus on transepithelial photorefractive keratectomy in China（2019）*”** *DOI: 10.3760/cma.j.issn.0412⁃4081.2019.03.003*

**Pre-operative** **use of medications**

Compound Tropicamide Eye Drops for cycloplegic refraction

0.5% levofloxacin eyedrops 4 times daily and bromfenac sodium eyedrops 3 times daily for anti-infection and anti-inflammatory

**Po-operative use of medications**

1. Immediately to 3 days after surgery

Bilateral bandage contact lenses, bromfenac sodium eye drops 2 times daily, tobramycin dexamethasone eye drops 4 times daily

2. Corneal epithelium healing and corneal bandage lens removal

Loteprednol etabonate ophthalmic suspension 3 times daily within 1month, 0.1% flurometholone eyedrops 3 times daily and decreasing once a month.

0.3% sodium hyaluronate eyedrops

carbomer eye gel 1 time at night

deproteinized calf blood extract eye gel 3 times daily

***Medication guidance from “Chinese expert in perioperative medication for laser corneal refractive surgery Consensus (2019)”*** *DOI: 10.3760/cma.j.issn.0412-4081.2019.12.006*
